# The safety of dental care for older adults during COVID-19 pandemic era

**DOI:** 10.1186/s13037-021-00300-x

**Published:** 2021-10-03

**Authors:** Ramya Shenoy, Violet D’Souza, M. Roma

**Affiliations:** 1 Department of Public Health Dentistry, Manipal College of Dental Sciences, Mangalore, Manipal Academy of Higher Education, Manipal, India; 2 Faculty of Dentistry, Université de Montréal, C.P. 6128, succ. Centre-ville, Montreal, QC H3C 3J7 Canada; 3 Department of Conservative Dentistry and Endodontics, Manipal College of Dental Sciences, Mangalore, Manipal Academy of Higher Education, Manipal, India

COVID-19 has paralyzed the world as its rampant spread has claimed over 162,773,940 confirmed cases and 3,375,573 lives worldwide as of 12 May,2021 since December 2019 [1]. This far-reaching effect is because COVID-19 gets transmitted through air bound droplets and human to human contact [2].

## How has this affected dental care?

Dental care involves generation of aerosol, splatter, and face to face interactions. These factors make dental care providers and patients susceptible to contracting COVID-19 [3].

As a result, dental care has been limited to emergencies only [4]. Use of teledentistry and video calls are becoming exceedingly popular as it reduces probability of spread of the virus [2].

## Why dental care is important for the older adults (over 65 years)?

Given that the older adults often have multiple comorbid conditions, they are susceptible for contracting nosocomial infections. Given their health condition, they may often require dental care at the hospitals or at special are centers and visiting the dentist only when there is pain and discomfort exposes older adults to an increased risk of infection due to long appointments or unavoidable use of rotary instruments. Therefore, it is important to take extra precautions and prevent the risk of spreading the virus to them.

Compromised chewing performance, limited food choice, weight loss, impaired speech, low self-esteem and wellbeing are some of but not limited to the effects of poor oral health in older adults [5].

## Adapting new strategies in dental care to help the older adults

Preventing older adults from contracting nosocomial infections is the challenge faced in geriatric dental care currently as underlying concomitant disorders make them susceptible. Literature suggests that, the dental care providers should adopt certain extra strategies for their own protection against infection [2].

Use of telecommunication and pre check triaging of the patients could be a valuable method to screen patients. Dental care providers have to prescribe chest CT scans and RT-PCR test for suspected patients or refer to physicians, can prevent them visiting the dental clinics [2]. Also installation of high volume suction, HEPA filters, UV filters and having negative pressure rooms to clear aerosols in the treatment room must be considered. Disinfection of the room and dental unit water lines’ as per American Dental Associations guidelines is a must [3].

Use of extra oral radiographs for diagnosis purposes, pre procedural mouth rinses, non-rotary hand instruments, chemo mechanical agents, cariostatic restoration materials and minimizing use of three way syringes are being encouraged [2, 3]. As aerosols remain hung in the air for over 24 h, some literature has suggested that treatments generating aerosols should be scheduled at the end of the day. For example, oral prophylaxis using scalers and curettes, steps involved in denture procedures, partial excavation of caries with slow speed hand piece and crown preparations can be scheduled as the last appointment in the day [4–6].

Dentists need to do, more than ever to stress on preventive care especially in the older adults, because visiting the dentist only when there is pain and discomfort exposes older adults to an increased risk of infection due to long appointments or unavoidable use of rotary instruments [4]. For the wellbeing of older adults, the medical and dental fraternities must work together to create awareness and formulate a preventive treatment plan to minimize the need for drastic dental intervention thereby reducing risk of contracting infectious diseases, like COVID-19.

## Conclusion

- Patients can opt for teledentistry to avail the initial visit to the dentist to prevent the spread of COVID-19.
- Based on the urgency of the situation, the dentist can render treatment for the older adults, whilst following all the infection control protocols and measures.
- In these COVID times, the older patients should maintain their oral hygiene by following simple practices of tooth brushing and regular oral rinses.
- To formulate a preventive treatment plan to minimize the need for drastic dental intervention thereby reducing risk of contracting infectious diseases, like COVID-19.

## Data Availability

Data sharing is not applicable to this article as no datasets were generated or analyzed during the current study.

## Abbreviations

COVID-19: Coronavirus disease
CT: Computed Tomography
RT-PCR: Reverse Transcriptase—Polymerase Chain Reaction
HEPA: High Efficiency Particulate Air
UV: Ultraviolet
